# Colored Point Cloud Completion for a Head Using Adversarial Rendered Image Loss

**DOI:** 10.3390/jimaging8050125

**Published:** 2022-04-26

**Authors:** Yuki Ishida, Yoshitsugu Manabe, Noriko Yata

**Affiliations:** 1 Graduate School of Science and Engineering, Chiba University, Chiba 263-8522, Japan; 2 Graduate School of Engineering, Chiba University, Chiba 263-8522, Japan; yata@chiba-u.jp

**Keywords:** point cloud completion, colored point cloud, deep learning

## Abstract

Recent advances in depth measurement and its utilization have made point cloud processing more critical. Additionally, the human head is essential for communication, and its three-dimensional data are expected to be utilized in this regard. However, a single RGB-Depth (RGBD) camera is prone to occlusion and depth measurement failure for dark hair colors such as black hair. Recently, point cloud completion, where an entire point cloud is estimated and generated from a partial point cloud, has been studied, but only the shape is learned, rather than the completion of colored point clouds. Thus, this paper proposes a machine learning-based completion method for colored point clouds with XYZ location information and the International Commission on Illumination (CIE) LAB ($L^{*}a^{*}b^{*}$) color information. The proposed method uses the color difference between point clouds based on the Chamfer Distance (CD) or Earth Mover’s Distance (EMD) of point cloud shape evaluation as a color loss. In addition, an adversarial loss to $L^{*}a^{*}b^{*}\text{-}Depth$ images rendered from the output point cloud can improve the visual quality. The experiments examined networks trained using a colored point cloud dataset created by combining two 3D datasets: hairstyles and faces. Experimental results show that using the adversarial loss with the colored point cloud renderer in the proposed method improves the image domain’s evaluation.

## 1. Introduction

Currently, three-dimensional (3D) data measurement is becoming prevalent with the development of depth sensors such as LiDAR sensors. Smartphones are also equipped with depth sensors for face authentication, portrait photography, and augmented reality (AR) applications. Point cloud data, the same as 3D data measurement and utilization, are becoming increasingly important and popular as a method of expression for 3D contents such as AR and virtual reality (VR). The human head is an important part of the human body for communication, and its 3D data can be used in communication as well. However, when a dark hair color is present, a single RGBD camera is prone to occlusion and depth measurement failure. Therefore, obtaining complete 3D data of the entire head from RGBD images of the head with depth loss is difficult.

With the advancement of machine learning-based methods in recent years, research on point clouds based on deep learning, which directly processes 3D point clouds, has advanced. One such task is called point cloud completion, where an entire point cloud is estimated and generated from an incomplete point cloud. Point cloud completion methods enable the completion of a single depth image with occlusion, obtaining an entire point cloud of the target object. However, previous studies on point cloud completion have only dealt with shapes and could not process point clouds, including color information.

Therefore, this paper proposes a machine learning-based colored point cloud completion method to obtain complete 3D data from RGBD images of the human head with depth loss in a section of a hair. As the loss for learning colored point clouds, this paper proposes a color difference loss between point clouds based on the Chamfer Distance (CD) or Earth Mover’s Distance (EMD), which are used for point cloud shape evaluation. In addition, to improve the visual quality, the proposed method applies an adversarial loss to $L^{*}a^{*}b^{*}\text{-}Depth$ images of the output point cloud from a differentiable point renderer. Experiments were conducted using the proposed method to complement a partial point cloud of the human head to a complete point cloud of the head, including color information, and the results were evaluated in the point cloud and image domains.

## 2. Related Work

3D measurement methods are mainly classified into two types: active and passive. The active type includes active stereo methods, which use a spotlight or pattern light, and optical radar methods, which measure light reflection. Light detection and ranging (LiDAR) and some RGBD cameras use time-of-flight sensors based on the optical radar method. It is challenging to use these active methods to measure the depth of an object that absorbs light because a light pulse is applied to the object, and the reflected light is read. The passive type includes stereo image methods, which calculate depth via triangulation using two cameras, and the Structure from Motion method, which calculates the camera position and 3D shape of an object from multiple images from different viewpoints. In these methods, it is necessary to search for the corresponding feature points from multiple images, and it is difficult to measure the 3D shape of an area with no pattern. Thus, it is difficult to measure the 3D shape of an object that absorbs light and whose features are difficult to detect. Therefore, hair color is typically monochromatic; depth measurement is likely to fail for dark hair such as black hair.

Recently, research on deep learning-based methods has advanced in various fields, including image processing, natural language processing, and audio processing. Various deep learning-based methods have been proposed for point cloud data, including PointNet [1]. PointNet is a classifier and segmentation network that inputs and outputs point cloud data directly. These point cloud data are unordered without any relationship among data points. In PointNet, a shared multilayer perceptron (MLP) is performed for each data point, and then max pooling is performed to extract features of the entire point cloud to achieve order invariance. In PointNet++ [2], point clouds are sampled and grouped in a multi-step process, and PointNet is applied to them to process local features. It also performs multiscale grouping, which combines the features of multiple scales. Dynamic graph convolution neural networks (CNNs) (DGCNN) [3] perform graph convolution on data points in a k-neighborhood in feature space. Point-voxel CNNs (PVCNNs) [4] are networks using PVConv, a combination of point cloud and voxel-based methods. PVConv internally voxelized and convoluted point clouds without increasing the resolution of voxels using a 3DCNN. Compared with PointNet++ and DGCNN, PVCNN achieves high accuracy in part segmentation with low latency and low graphics processing unit (GPU) memory usage.

FoldingNet [5] is an autoencoder for point cloud shapes. The decoder of FoldingNet does not transform into a 3D point cloud using the fully connected layer but instead transforms the two-dimensional grid coordinates into the point cloud surface by folding them step by step.

The point completion network [6] is a point cloud completion method and is a PointNet based encoder with shared MLP and max pooling. Its decoder is a multi-stage generator that generates a coarse output point cloud using a fully connected layer and then outputs a detailed point cloud using a folding-based layer. The morphing and sampling network (MSN) [7] uses a morphing-based decoder. MSN uses a folding-based method to generate multiple patch point clouds for the middle output. Thus, it is possible to deal with complex shapes that are difficult to achieve by folding a single grid. Then, the middle output point cloud is combined with the input point cloud and downsampled to uniform density by minimum density sampling. Finally, the final point cloud is output by refinement using a residual network. Since the EMD is a better visual metric than the CD, which is often used to evaluate a point cloud shape, MSN uses an approximate algorithm EMD for training. SpareNet [8] uses channel-attentive EdgeConv in its encoder and residual network. The morphing-based decoder uses style-based point generation, which is inspired by Style-Generative Adversarial Network(GAN) [9]. Additionally, a differentiable point renderer converts an output point cloud into a depth image, and a CNN-based discriminator is used for adversarial learning. There is Cycle4Completion [10], which establishes a geometric correspondence between complete and incomplete shapes from both directions rather than one direction from incomplete shapes to complete shapes. The other point cloud completion method uses the adversarial samples with PMPD-k attack (k-iteration projection on the mean of principal directions attack) [11].

Point2Color [12] performs point cloud colorization to estimate the color information of each point from a point cloud with no color information. The network uses PointNet++ for coloring. For training, Point2Color applies adversarial loss to the output point cloud using PointNet++ and adversarial loss to the rendered image of the output point cloud using CNNs.

## 3. Proposed Method

### 3.1. Dataset

Deep learning requires a large amount of data for training. However, there is no large-scale 3D dataset of the entire heads, including hair. This study combines a human face 3D dataset and a hairstyle dataset to create a head point cloud dataset, including color information.

This study uses FaceScape [13] as the face dataset. FaceScape contains 847 faces, with 20 different facial expressions recorded for each face. Five expressions from the dataset are used: neutral, mouth stretch, jaw left, lip roll, and eyes closed.

This study uses USC-HairSalon [14] as a hairstyle dataset. USC-HairSalon contains 343 types of hairstyles. The same data expansion is performed as in HairNet [15], one of the previous studies using USC-HairSalon. In this data extension, each hairstyle is classified by hair length (XS, S, M, L, and XL) and hair curl (straight or curly), and two hairstyles of the same type can be blended to create a new hairstyle.

The 3D data of these faces and hairstyles are combined using the 3DCG software Blender [16].

The Particle Hair and the Principal Hair BSDF shader are used to draw the hair. Melanin and Roughness are set randomly to reproduce black, brown, and blonde hair, rendering RGBD images. A single point cloud is created as the ground truth point cloud based on RGBD images from six directions. This point cloud is downsampled and converted to a point cloud of 16,384 data points.

**Figure 1:**
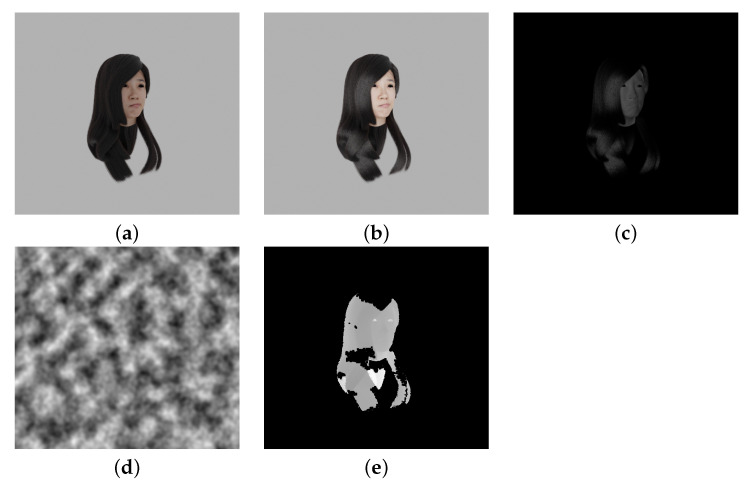
Reproduction of missing depth images in input point clouds. (**a**) color image; (**b**) lighted image; (**c**) difference image; (**d**) Perlin noise; (**e**) reproduced missing depth image.

For partial input point cloud, we created RGBD images by placing cameras at 60${}^{{}^{\circ}}$ left and right, 30${}^{{}^{\circ}}$ above, and 10${}^{{}^{\circ}}$ below the center of the neck randomly. The amount of change when the hair is illuminated and the Perlin noise to reproduce the depth loss in the hair part are used, as shown in Figure 1. This study assumes that the part of the hair that tends to have depth loss in the dark hair color, such as black hair, is the part that absorbs light. In this study, we calculated the difference in grayscale between the original image and an image illuminated from the camera position and deletes the depth information in areas in which the difference is small. The depth image of the hair part is further masked using Perlin noise. This RGBD image is converted to a point cloud and downsampled to data 4096 points. These input point clouds are created from four different directions for one ground truth point cloud.

**Figure 2:**
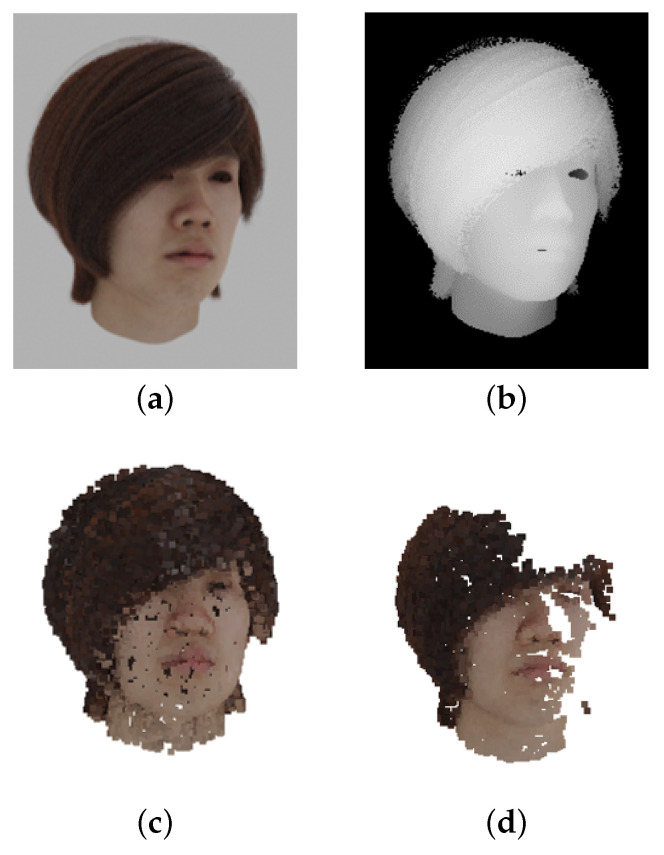
An example of a colored point cloud data set of the head. (**a**) color image; (**b**) depth image; (**c**) ground truth point cloud; (**d**) partial input point cloud.

Finally, the dataset is scaled in this study so that the 3D position coordinates are in the range of [−1,1]. In addition, the color information is converted from RGB to $L^{*}a^{*}b^{*}$ and multiplied by 1/100. OpenCV [17] is used for color transformation, and the white point is D65. An example of the created colored point cloud dataset is depicted in Figure 2. The dataset was created by dividing the original data into 70/15/15 as training/validation/test sets.

### 3.2. Network

**Figure 3:**
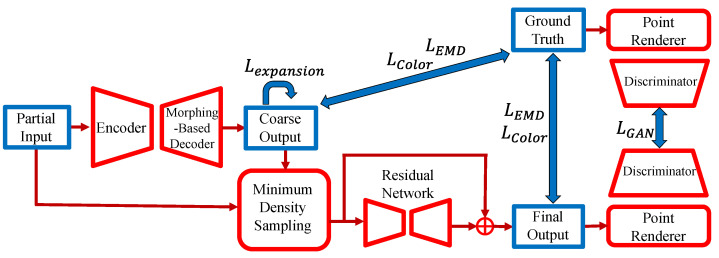
Architecture of the proposed network.

A network based on the MSN [7] architecture is used, as shown in Figure 3. Preliminary experiments with point completion network [6] resulted in more approximate output. Therefore, an MSN based network with a refiner that adds the input and coarse outputs together and processes them again is adopted from previous studies of point cloud completion. There is also the issue of computational complexity, since the network has an encoder, decoder, refiner, point renderer, and discriminator.

After the encoder extracts global features, the morphing-based decoder morphs the 32 unit squares [0,1] into a patch point cloud of 512 data points and outputs a coarse point cloud of 16,384 data points. The middle output point cloud and the input point cloud are added and converted into a point cloud of 16,384 data points with uniform density using minimum density sampling. Finally, a residual network is used to refine and output the final point cloud. In MSN, only XYZ position coordinates are inputs and outputs. This study inputs and outputs the point data of six dimension vectors, including $L^{*}a^{*}b^{*}$ color information, and complements the colored point cloud. Since the $L^{*}a^{*}b^{*}$ color space is a uniform color space, unlike the RGB color space, it can approximate the color difference perceived by humans using the Euclidean distance.

**Figure 4:**
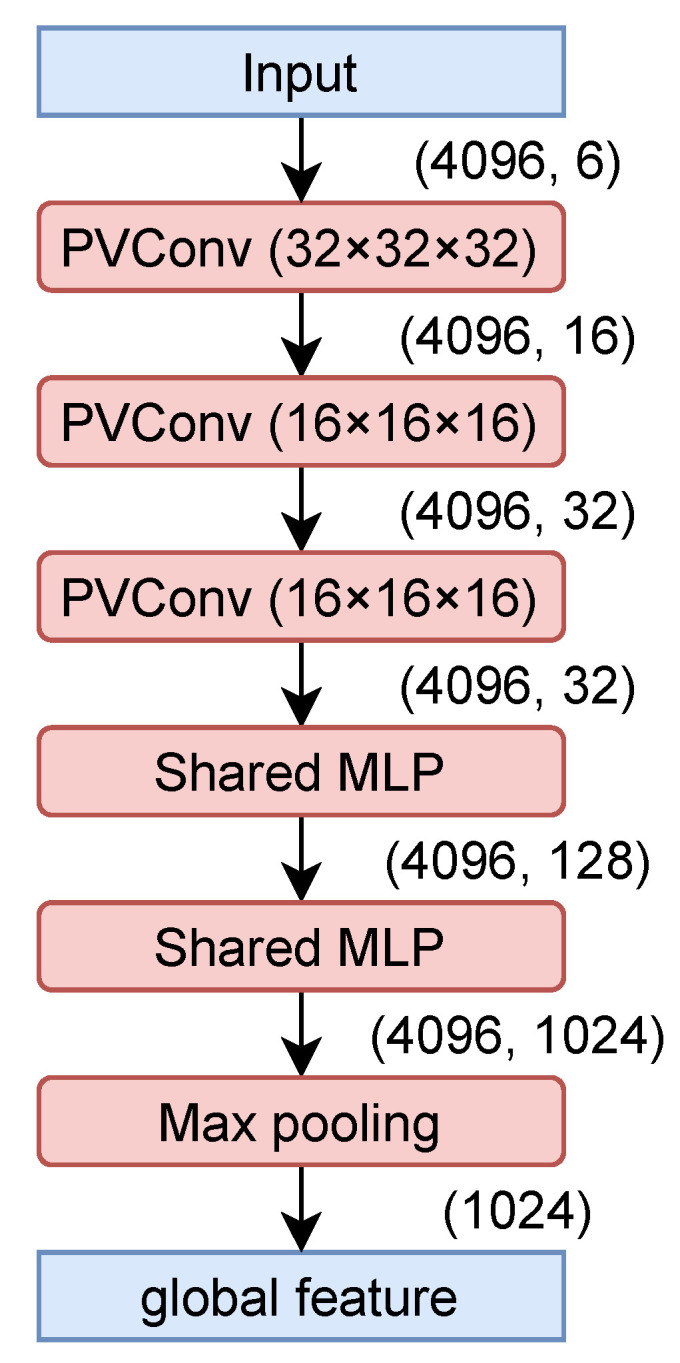
Encoder of the proposed network.

**Figure 5:**
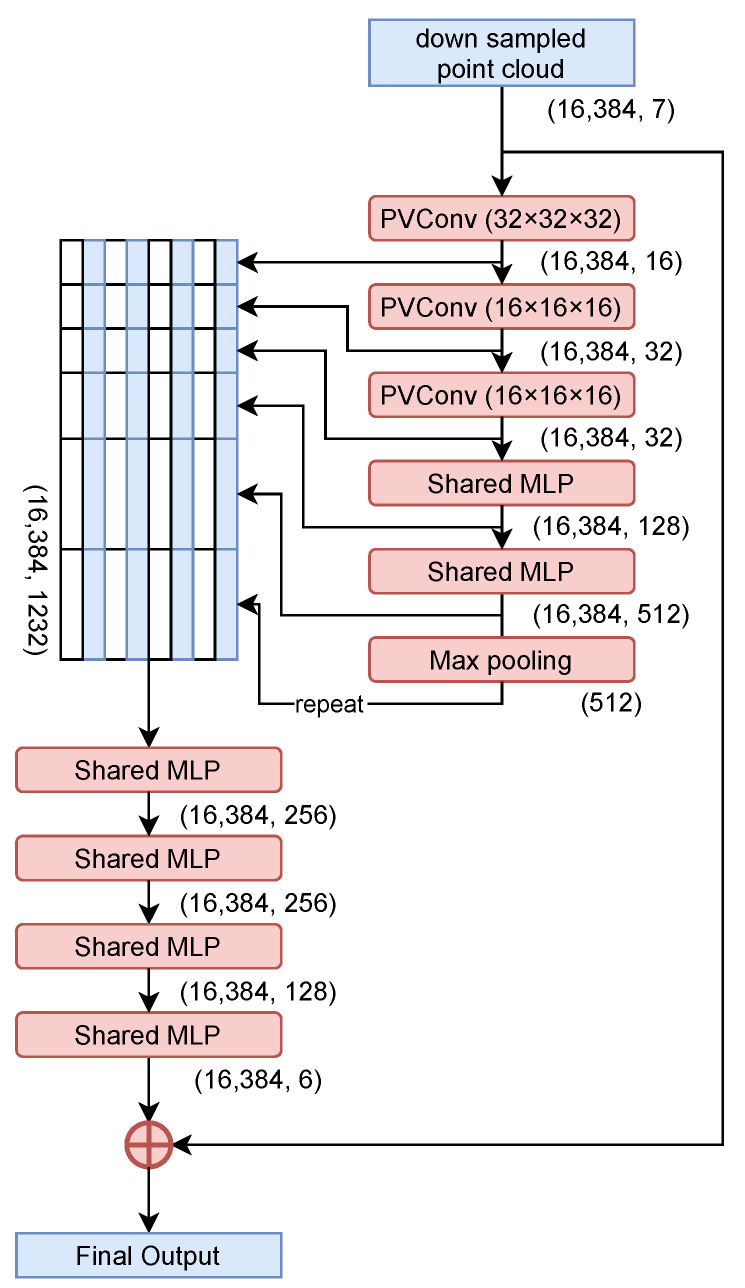
Residual network of the proposed network.

In the proposed network, the encoder and residual network are changed to use PVConv [4]. As shown in Figure 4 and Figure 5, global features and difference information are extracted by convolution using PVConv with voxel resolutions of 32×32×32 and 16×16×16. As the decoder, SpareNet’s [8] style-based folding layer is used.

**Figure 6:**
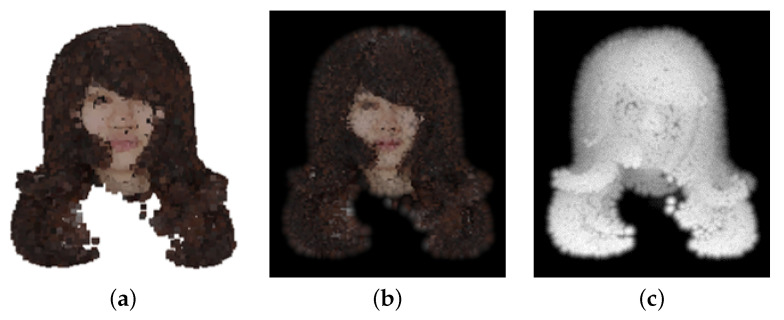
Point-rendered image of a point cloud; (**a**) point cloud; (**b**) color image; (**c**) depth image.

A GAN with a differentiable point renderer is used for training. This network is based on the SpareNet implementation but modified to render $L^{*}a^{*}b^{*}\text{-}Depth$ images, as shown in Figure 6. The point renderer is used to create $L^{*}a^{*}b^{*}\text{-}Depth$ images of the input and output/ground truth point cloud from four directions: front, back, left, and right. The discriminator primarily consists of four CNN blocks to determine whether an image is of the ground truth point cloud or the complemented point cloud.

### 3.3. Loss

CD [18] and EMD [18] are frequently used as evaluation methods for point cloud shapes. CD represents the average distance between each point in point cloud $S_{1}$ and the nearest neighbor in another point cloud $S_{2}$:

(1) $$L_{CD}\left(S_{1},S_{2}\right)=\frac{1}{2}(\frac{1}{|S_{1}|}\sum_{x\in S_{1}}min_{y\in S_{2}}||x-{y||}_{2}+\frac{1}{|S_{2}|}\sum_{x\in S_{1}}min_{y\in S_{1}}||x-{y||}_{2})$$

On the other hand, EMD is computed for two point clouds of the same size so that the average distance of bijection ϕ pairs is the smallest:

(2) $$L_{EMD}\left(S_{1},S_{2}\right)=\sum_{\phi :S_{1}\to S_{2}}\frac{1}{|S_{1}|}min_{x\in S_{1}}||x-\phi \left(x\right){\left|\right|}_{2}$$

As for MSNs, EMD is more computationally expensive but has better visual evaluation performance than CD. Therefore, EMD is used as the loss function of the point cloud shape using an approximation algorithm similar to MSN.

As the loss function for color, we used the average color difference in the $L^{*}a^{*}b^{*}$ color space between the points for which CD or EMD calculates the distance. In addition, the expansion loss $L_{exp}$, which suppresses the expansion of each patch point cloud by the morphing-based decoder, is added to the loss function. Without a GAN, the loss function consists of the shape loss $L_{EMD}$, color loss $L_{color}$ for the middle and final output point cloud, and expansion loss $L_{exp}$ for the middle output point cloud:

(3) $$\begin{array}{cc} L_{withoutGAN}= & L_{EMD}\left(S_{gt},S_{coarse}\right)+L_{EMD}\left(S_{gt},S_{final}\right)\\ \; & +\omega_{color}\left(L_{color}\left(S_{gt},S_{coarse}\right)+L_{color}\left(S_{gt},S_{final}\right)\right)+\omega_{exp}L_{exp}\left(S_{coarse}\right) \end{array}$$

The weights are set to $\omega_{color}=0.3$ and $\omega_{exp}=0.1$.

In the case of using a GAN, $L_{withGAN}$ adds an adversarial loss $L_{GAN}$ to $L_{withoutGAN}$. $L_{GAN}$ consists of the loss $L_{adv}$ for estimating the $L^{*}a^{*}b^{*}\text{-}Depth$ image π(S) using the discriminator, the L-1 distance $L_{img}$ between $L^{*}a^{*}b^{*}\text{-}Depth$ images of the output point cloud and the ground truth point cloud, and the L-2 distance $L_{feat}$ between feature maps $D_{i}$ of complemented point cloud images and ground truth point cloud images using four blocks of the discriminator. These losses are formulated as

(4) $$L_{adv}={\left(1D\left[\pi \left(S_{final}\right)\right]\right)}^{2}$$

(5) $$L_{img}=\frac{1}{4HWC}||\pi \left(S_{gt}\right),\pi \left(S_{final}\right){\left|\right|}_{1}$$

(6) $$L_{feat}=\sum_{i}^{4}\frac{a_{i}}{H_{i}W_{i}C_{i}}\left|\right|D_{i}\left[\pi \left(S_{gt}\right)\right],D_{i}\left[\pi \left(S_{final}\right)\right]{\left|\right|}_{2}^{2}$$

(7) $$a_{i}=\frac{C_{i}}{\sum_{i}^{4}C_{i}}$$

with HWC as height, width, and channels of feature map shape by each deiscriminator block *i*.

(8) $$L_{withGAN}=\omega_{rec}L_{withoutGAN}+L_{GAN}$$

The weights are set to $\omega_{rec}=200.0$, $\omega_{adv}=0.1$, $\omega_{img}=1.0$, and $\omega_{feat}=1.0$.

## 4. Experimental Results

Four types of training were performed using MSNs with only input/output changes: CD/EMD-based color loss, with/without GANs. In this study, we also experimented with the proposed network for CD-based color loss, with/without GANs. Adam was used as the optimization function for the training. The training was performed for 150 epochs each, and the one with the lowest validation loss was adopted. Each training took approximately five days on a computer equipped with three RTX2080ti or GTX1080ti.

### 4.1. Morphing and Sampling Network

**Figure 7:**
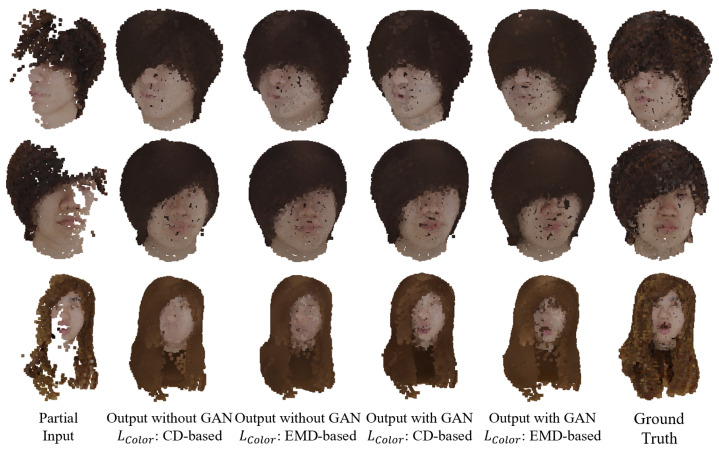
Completion results using MSN.

Figure 7 shows the result of the colored point cloud completion for the test data using the learned MSN. The figure shows that the point cloud of the entire head can be roughly generated from the missing point cloud, including color information. In the case with GAN, parts such as the mouth are drawn more clearly than in the case without GAN.

Table 1 shows the evaluation result of CD, EMD, CD-based color difference, EMD-based color difference, $L_{img}$, and Fréchet Inception Distance (FID) [19] for the trained MSN using the test data. CD and EMD are the most common metrics for evaluating point cloud shapes in point cloud completion tasks. FID measures the distance between the distributions of two image groups and is used in image generation tasks employing a GAN. In this experiment, the final output point cloud and the ground truth point cloud are evaluated as point-rendered images from the front. Table 1 shows that the accuracy of the point cloud shape tends to be higher when the EMD-based color difference loss is used. It is considered that the learning converged well because the calculations of the shape and the color difference were performed at the same points.

On the other hand, for the color difference between CD- and EMD-based point clouds, the accuracy tends to be higher when using the CD-based color loss. Therefore, the CD-based color loss is considered appropriate. Since $L_{img}$ is the L1 distance to rendered $L^{*}a^{*}b^{*}\text{-}Depth$ images, models without a GAN with better CD, EMD, and color difference evaluation produced better results. In addition, when using a GAN, the evaluation in the point cloud domain is lower, but the evaluation in the FID is higher than that without a GAN. In other words, more visually appropriate completion is possible by using the adversarial loss with a differentiable point renderer.

### 4.2. Proposed Network

**Figure 8:**
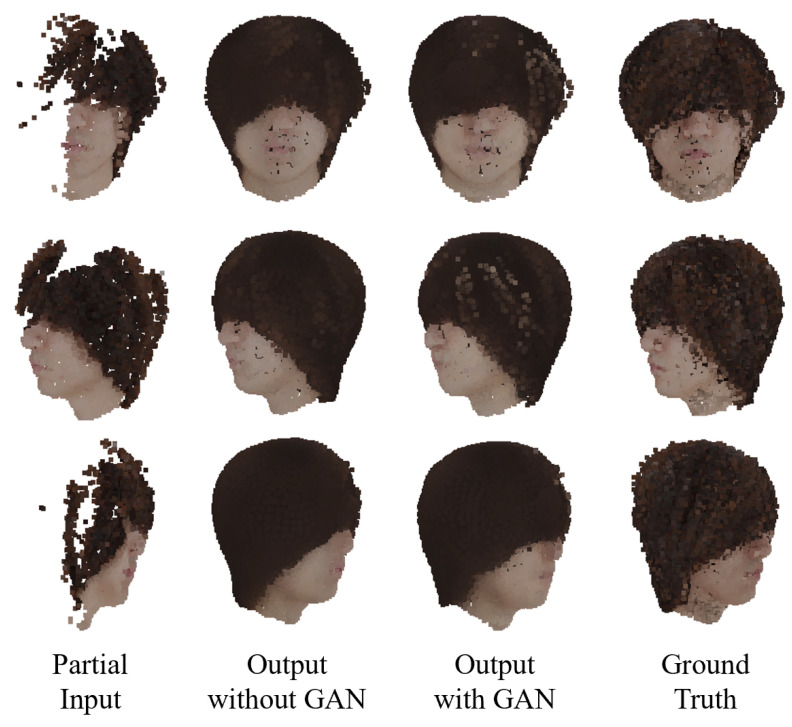
Completion example 1 using the proposed network.

**Figure 9:**
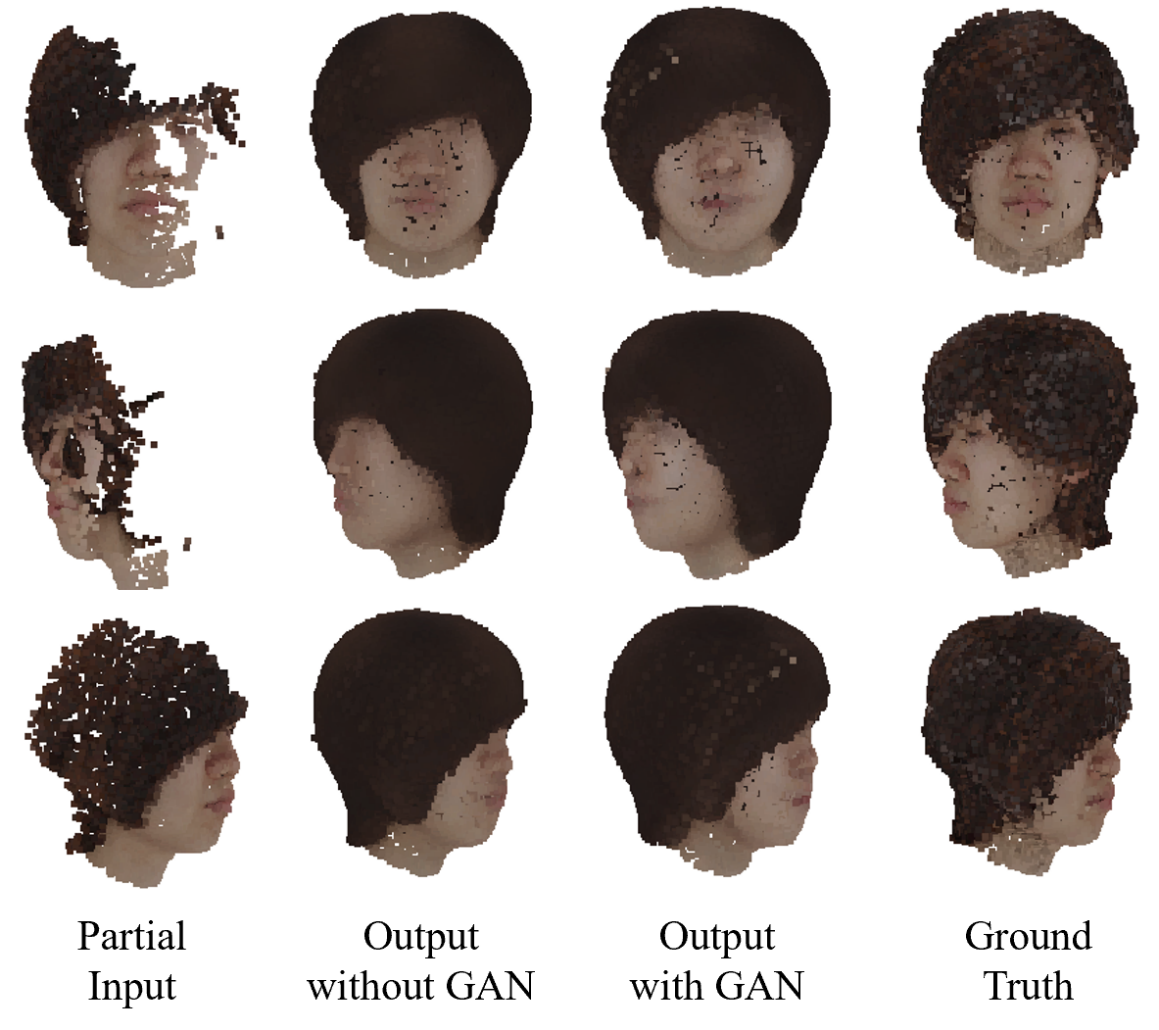
Completion example 2 using the proposed network.

**Figure 10:**
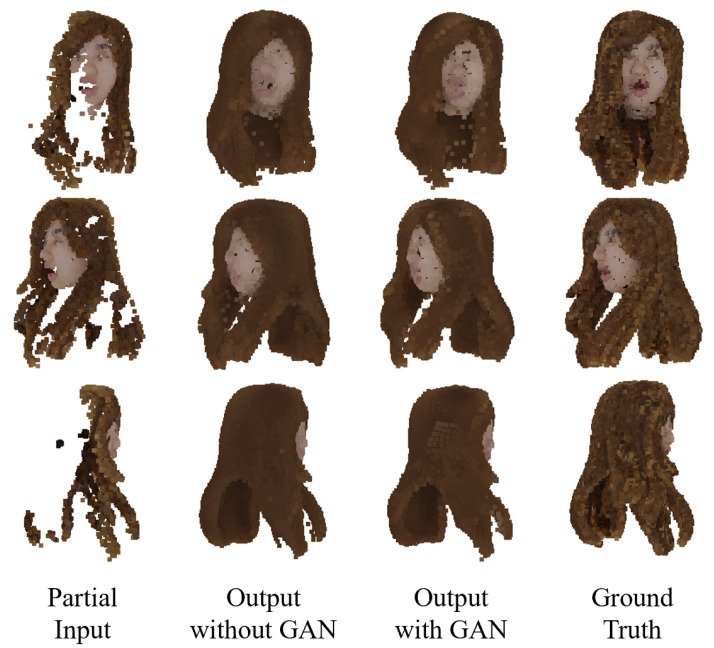
Completion example 3 using the proposed network.

**Figure 11:**
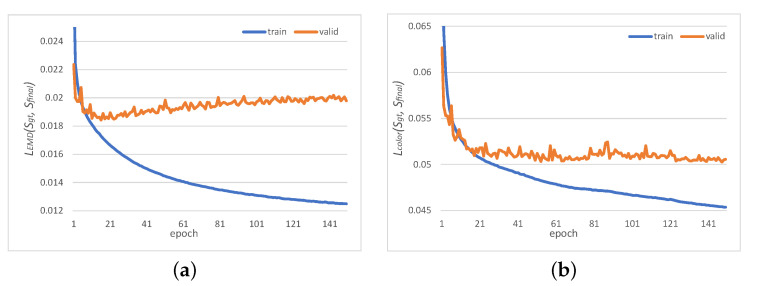
Losses during the learning. (**a**) EMD for final output; (**b**) CD-based color difference for final output.

Figure 8, Figure 9 and Figure 10 show the colored point cloud completion results for the test data using the trained proposed network. Comparing the evaluation result with the result of MSN using the same loss function in Table 2, the proposed network performed better in all metrics. However, in terms of the EMD, the proposed network could not outperform the MSN using the EMD-based color difference loss without a GAN. This is due to the proposed network’s overtraining, Figure 11 shows the trends of $L_{EMD}\left(S_{gt},S_{final}\right)$ and $L_{color}\left(S_{gt},S_{final}\right)$ during the training of the proposed network with a GAN. From Figure 11, the proposed network is overtrained, especially in terms of shape. It is necessary to expand the dataset to suppress overlearning.

Furthermore, interpolation did not work well for complex hair shapes such as long and curly hair. This appears to occur because the shape tends to be average and the point density is insufficient. For complex shapes, the use of an adversarial loss by a point cloud-based discriminator and upsampling of the point cloud by a multi-step decoder should be considered.

In addition, these results show that some details of the input point cloud may not be retained in the output. This is because the input point cloud is encoded. Increasing the size of global features may mitigate this problem, but we believe it is a tradeoff for overlearning. The proposed method uses a residual network for refinement, but it samples a point cloud regardless of whether it is an input point cloud or a coarse output point cloud, so some details may not be retained. In the future, we will consider using refinement twice, as SpareNet does, or using skip connections to convey input details other than global features. Furthermore, adding a one-sided CD from the input point cloud to the loss function may mitigate this problem.

### 4.3. Completion of Actual Data

**Figure 12:**
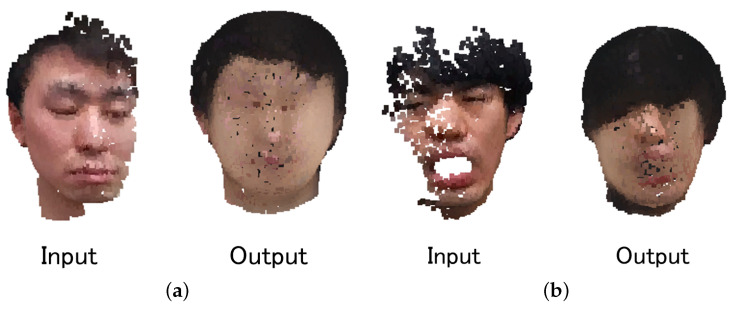
Results of completion the actual data using the trained proposed network with GAN. (**a**) Completion example 1; (**b**) Completion example 2.

Figure 12 shows the completion result of the actual data measured by Azure Kinect DK using the proposed network with a pretrained GAN. This result confirmed that the color of the face part was not reproduced correctly compared with the complete results for the test data. This is probably because the lighting environment of all data in the training dataset was the same. Therefore, it is necessary to create a dataset reproduced in various lighting environments in the future.

## 5. Conclusions

In this study, we proposed a machine learning-based completion method for colored point clouds, which complements a colored point cloud with missing head parts to a complete point cloud of the entire head, including color information. We demonstrated that an approximate shape can be obtained using a dataset created by combining 3D data of the face and hair. Colored point clouds can be learned by using a color difference loss based on the CD or EMD. For the evaluation of color and images, we found that using the CD-based color loss is better than the EMD-based color loss. In addition, the proposed network improved the completion accuracy by using PVConv and the style-based folding layer. In this study, we confirmed that learning with the adversarial loss for $L^{*}a^{*}b^{*}\text{-}Depth$ images using a point renderer provides adequate visual completion.

Future work includes improving the network and loss function and expanding the dataset. The current method cannot adapt complex hairstyles and data measured with actual RGBD cameras, and the point density is insufficient to represent details. Methods that have proven effective in point cloud completion, such as a multi-step generator and a skip connection, could be used to improve the process. It is necessary to compare how these point cloud completion methods differ in learning colored point clouds. Adding a point cloud-based adversarial loss and a one-sided CD from input point clouds to the loss function is expected to realize better desirable learning. The dataset also needs to be expanded to reproduce various measurement environments to mitigate overfitting and improve real data completion. The completion output of our network tends to be blurry, and the completion accuracy needs to be improved for practical applications. We believe that deep learning-based colored point cloud completion can be extended not only to the head but also to other types of input, such as the human body. It is expected that the colored point cloud completion will be supplemented to the head and the entire body in the future and will apply to several scenes.

## Figures and Tables

**Table 1:** Evaluation results of learning with MSN.

	CD	EMD	CD-Based Color Difference	EMD-Based Color Difference	$L_{\mathrm{img}}$	FID
$L_{color}$: CD-based, $L_{GAN}$: unused	1.464 × $10^{-2}$	4.367 × $10^{-2}$	5.176 × $10^{-2}$	5.427 × $10^{-2}$	6.830 × $10^{-3}$	127.8
$L_{color}$: EMD-based, $L_{GAN}$: unused	1.443 × $10^{-2}$	3.992 × $10^{-2}$	5.296 × $10^{-2}$	5.465 × $10^{-2}$	6.805 × $10^{-3}$	122.8
$L_{color}$: CD-based, $L_{GAN}$: used	1.549 × $10^{-2}$	4.478 × $10^{-2}$	5.281 × $10^{-2}$	5.522 × $10^{-2}$	7.126 × $10^{-3}$	94.7
$L_{color}$: EMD-based, $L_{GAN}$: used	1.494 × $10^{-2}$	4.441 × $10^{-2}$	5.711 × $10^{-2}$	5.736 × $10^{-2}$	7.163 × $10^{-3}$	100.7

**Table 2:** Evaluation results of learning with the proposed network.

	CD	EMD	CD-Based Color Difference	EMD-Based Color Difference	$L_{\mathrm{img}}$	FID
$L_{color}$: CD-based, $L_{GAN}$: unused	1.352 × $10^{-2}$	4.172 × $10^{-2}$	4.986 × $10^{-2}$	5.251 × $10^{-2}$	6.504 × $10^{-3}$	110.2
$L_{color}$: CD-based, $L_{GAN}$: used	1.345 × $10^{-2}$	4.139 × $10^{-2}$	5.023 × $10^{-2}$	5.321 × $10^{-2}$	6.550 × $10^{-3}$	80.9

## Data Availability

Redistribution of FaceScape [13] and USC-HairSalon [14] is prohibited. Please contact them directly. Other data that support the findings of this study are available from the corresponding author, upon reasonable request.
